# Aging: Mitigation and Intervention Strategies

**DOI:** 10.1155/2015/276539

**Published:** 2015-03-10

**Authors:** Chi-Feng Hung, Jorge Azofeifa-Navas, Huanran Tan, Chih-Chi Andrew Hu, Nicole Clarke

**Affiliations:** ^1^School of Medicine, Fu Jen Catholic University, New Taipei City 24205, Taiwan; ^2^School of Biology, University of Costa Rica, 11501-2060 San José, Costa Rica; ^3^University Health Science Center, 38 Xueyuan Road, Beijing 100191, China; ^4^Moffitt Cancer Center, Tampa, FL 33612, USA; ^5^University of Nottingham, Nottingham NG7 2RD, UK

Aging is a natural process of life. The causes of aging may come from different sources, both internally and externally. Cellular aging may lead to organ dysfunction during this complicated, irreversible process. Shortening of telomeres during each cell division may lead to cell death; a cell stops dividing while its telomeres shrink to a critical minimum size; and it is one of the causes found in the degeneration of our vision, memory, and immune system. Another contributor to the aging process is oxidative stress. Increased reactive oxygen species (ROS) levels caused by environmental pollution, ionizing radiation, food additives, and so forth may mutate our genes; induce harmful effects on our cells, such as oxidations of lipid, amino acids in proteins, and inactivate specific enzymes; and be a causal factor of cancer development and premature aging. In order to live healthily while we grow old, healthy eating and life style are crucial. Meanwhile, scientists spend a lot of effort trying to investigate ways to support healthy aging and prevent or delay the onset of age-related disease and decline. Many natural ingredients are also found to be beneficial; for example, flavonoids may help fight oxidative stress.

This special issue was to collect information on the mechanism of aging and idea on how to mitigate, delay, or treat age-related decline and diseases. The issue includes studies particularly focusing on the roles of ROS and inflammation and gene in aging and antiaging, as well as investigations of natural products and medications related to antiaging, anticancer, and prevention of cardiovascular diseases, thus promoting a healthy aging from different aspects.

*Chi-Feng Hung* *Jorge Azofeifa-Navas* *Huanran Tan* *Chih-Chi Andrew Hu* *Nicole Clarke*

